# From DNA Damage to Cancer Progression: Potential Effects of Cytolethal Distending Toxin

**DOI:** 10.3389/fimmu.2021.760451

**Published:** 2021-11-15

**Authors:** Yi-Ru Lai, Yu-Fang Chang, Jason Ma, Cheng-Hsun Chiu, Ming-Ling Kuo, Chih-Ho Lai

**Affiliations:** ^1^ Graduate Institute of Biomedical Sciences, College of Medicine, Chang Gung University, Taoyuan, Taiwan; ^2^ Department of Microbiology and Immunology, College of Medicine, Chang Gung University, Taoyuan, Taiwan; ^3^ Molecular Infectious Disease Research Center, Department of Pediatrics, Chang Gung Memorial Hospital, Linkou, Taiwan; ^4^ Division of Allergy, Asthma, and Rheumatology, Department of Pediatrics, Chang Gung Memorial Hospital, Linkou, Taiwan; ^5^ Department of Microbiology, School of Medicine, China Medical University, Taichung, Taiwan; ^6^ Department of Nursing, Asia University, Taichung, Taiwan

**Keywords:** bacterial genotoxin, cytolethal distending toxin, DNA damage, genotoxicity, cancer development

## Abstract

Cytolethal distending toxin (CDT), one of the most important genotoxins, is produced by several gram-negative bacteria and is involved in bacterial pathogenesis. Recent studies have shown that bacteria producing this peculiar genotoxin target host DNA, which potentially contributes to development of cancer. In this review, we highlighted the recent studies focusing on the idea that CDT leads to DNA damage, and the cells with inappropriately repaired DNA continue cycling, resulting in cancer development. Understanding the detailed mechanisms of genotoxins that cause DNA damage might be useful for targeting potential markers that drive cancer progression and help to discover new therapeutic strategies to prevent diseases caused by pathogens.

## Introduction

Bacterial genotoxins are toxins that trigger single-strand breaks (SSBs) or double-strand breaks (DSBs) on DNA in target host cells, and are functionally homologous to mammalian type I deoxyribonuclease (DNase I), resulting in the activation of DNA damage response (DDR) (1). These responses subsequently lead to cell senescence, apoptosis, or genomic instability, which favors tumor initiation and progression. Three bacterial virulence factors are now characterized as genotoxins: cytolethal distending toxin (CDT) in gram-negative bacteria, typhoid toxin produced by *Salmonella enterica* serovar Typhi, and colibactin produced by the phylogenetic group B2 *Escherichia coli* (2). CDT was discovered in *E. coli* by Johnson and Lior in 1987 (3), and similar toxin activities were found in two other enteric pathogens, *Shigella* spp (3). and *Campylobacter* spp (4). CDT is capable of modulating eukaryotic cell cycle by pausing the G2/M transition, and was thus further defined as cyclomodulins (5). To clarify the possible virulence factors in enteric pathogens, cloning and gene sequencing were performed within different strains of *E. coli* and three open reading frames (ORFs) in an operon were identified, known as *cdtA*, *cdtB*, and *cdtC* (6, 7). Among the proteins encoded by these genes, CdtB was demonstrated to harbor nuclease activity (8–10). CdtA and CdtC are required for delivering CdtB into target cells, which allows for CdtB to translocate into the nucleus and cause DNA damage (11, 12). The catalytic activity of CdtB in target cells can activate DDR, which increases genomic instability, disturbs the cell cycle, and establishes a chronic proinflammatory environment (13). Since these characteristics are closely associated with cancer development, it is proposed that bacterial infections play a role in the neoplastic process. This review highlights the current state of knowledge on the interaction of CDT with host DNA and its role in tumor progression.

## Bacterial Infections Induce Cancer Development

Cancer risk is generally attributed to hereditary, genetic, environmental, and lifestyle factors (14). The contribution of infectious agents to cancer development is often underappreciated. In fact, more than 16% of cancer cases are related to infectious agents worldwide (15). Persistent infection-induced chronic inflammation, which is likely to be associated with the secretion of virulence factors, ultimately facilitates oncogenic processes in hosts (16–18). Bacterial toxins disrupt cellular signals, including cell proliferation, cell cycle progression, and DNA repair, and dysregulation of either of which is intimately intertwined with oncogenesis (19). For instance, *Helicobacter pylori*, remaining the most notorious pathogen to cause cancer, has been identified as a group 1 carcinogen by the International Agency for Research on Cancer since 1994 (20). It can secrete cytotoxin-associated gene A (CagA), which empowers cells with numerous cancerous traits, including cell death resistance, adherence junctional defects, and genomic instability after its entry into gastric epithelial cells *via* type IV secretion system (20). In addition, *Salmonella* species produce AvrA protein, a deubiquitinase that inhibits β-catenin ubiquitination to promote colonic epithelial cell proliferation (21). Enterotoxigenic *Bacteroides fragilis* secretes *B. fragilis* toxin (BFT), a zinc-dependent metalloprotease that can induce colitis and colorectal cancer (CRC) in multiple intestinal neoplasia (Min) mice (22, 23). Notably, with the growing number of studies on microbiota, genotoxin-producing bacteria have also been identified as potential carcinogens (24–27). In addition, other gastrointestinal tract-dwelling pathogens reported to produce CDT, including *C. jejuni* and *Helicobacter hepaticus*, may also have an impact on carcinogenesis (28–31). Collectively, the above studies indicate that bacteria together with their virulence factors not only cause infectious diseases, but also promote cancer development (**Table 1**). Therefore, the mechanisms involved in cancer development caused by bacteria and their toxins deserve further investigation.

**Table 1:** The relationships between bacterial pathogens, virulence factors, and cancers in the animal models.

Bacterium (toxin)	Related cancer	Animal model
***C. jejuni* (CdtB)**	Colorectal cancer	Germ-free *Apc^Min/+^*/DSS mice (28)
**CoPEC (Colibactin)**	Colorectal cancer	*Apc^Min/^* ^+^ mice (24, 25) AOM–treated *Il10^−/−^* mice (26) *Apc^Min/^* ^+^; *Il10^−/−^* mice (27)
***E. coli***	Prostate cancer	PhIP-treated mice (32)
**ETBF (BFT)**	Colorectal cancer	*Apc^Min/^* ^+^ mice (22)
***F. nucleatum***	Breast cancer	Orthotropic AT3 C57BL/6 mice (33)
***H. hepaticus* (CDT)**	Hepatocellular carcinoma	A/JCr mice (34)
***H. hepaticus* (CDT)**	Intestinal carcinoma	129/SvEv *Rag2* ^−/−^ mice (35)
***H. pylori* (CagA)**	Gastric adenocarcinoma	CagA transgenic mice (36)
***H. pylori* (CagA)**	Intestinal adenocarcinoma Small cell carcinoma	CagA transgenic zebrafish with p53 loss (37)
***P. gingivalis*** ***F. nucleatum***	Oral squamous cell carcinoma	4NQO-treated mice (38)
***Salmonella* (AvrA)**	Colorectal cancer	AOM/DSS-treated mice (39)

4NQO, 4-nitroquinoline-1-oxide; AOM, azoxymethane; BFT, B. fragilis toxin; CoPEC, colibactin-producing E. coli; DSS, dextran sulfate sodium; E. coli, Escherichia coli; ETBF, enterotoxigenic Bacteroides fragilis; F. nucleatum, Fusobacterium nucleatum; H. hepaticus, Helicobacter hepaticus; H. pylori, Helicobacter pylori; Min, multiple intestinal neoplasia; P. gingivalis, Porphyromonas gingivalis; PhIP, 2-amino-1-methyl-6-phenylimidazo[4,5-b]-pyridine.

## Bacterial Genotoxins and Their Biological Functions

Among the bacterial genotoxins, CDT is the first to be characterized and shown to cause DSBs (8, 40). In most CDT-harboring bacteria, the gene cluster encoding CDT subunits, consisting of adjacent or slightly overlapping *cdtA*, *cdtB*, and *cdtC*, is located on the chromosome (41). Special exceptions occur in some *E. coli* strains, in which the operon is found on a large conjugative plasmid called pVir (42). The location of the *cdt* cluster differs in the genomes of different species but is well conserved within the same species (43). In most cases, the expression of all three genes is indispensable for CDT toxicity (44), although the identified *cdt* is mainly composed of three ORFs (*cdtA*, *cdtB*, and *cdtC*), apart from *Salmonella enterica* serovar Typhi (*S.* Typhi). Notably, *S.* Typhi *cdt* contains a conserved *cdtB*, whereas *cdtA* and *cdtC* are substituted by genes encoding two homologs of the pertussis toxins, referred to as pertussis-like toxins A and B (PltA and PltB) (45).

Studies have analyzed the prevalence of CDT production in different bacterial species, including *C. jejuni*, *A. actinomycetemcomitans*, *H. ducreyi*, etc., from clinical specimens, and revealed that the majority of these species produce stable amounts of CDT (46–49). CDT is a prominent virulence factor of CDT-producing bacteria and aids in effective tissue colonization, thereby promoting potent infection by breaking down host defense (50). The dampened host defense mainly results from: (i) disrupted epithelial barrier, which is caused by CDT-induced cell cycle arrest and subsequent cell death in epithelial cells (51); and (ii) impaired host immunity, which is caused by the extreme sensitivity of lymphocytes to CDT cytotoxicity and altered macrophage functions (52, 53). To perform such sophisticated tasks, the CDT must first enter the cells to exert its activity. CDT holotoxin contains one active subunit (CdtB), which requires two binding subunits (CdtA and CdtC) to facilitate its transport through the cell membrane (54). The homology of CDT subunits varies among different bacterial species, and the pairwise identity of CdtA and CdtC ranges from 19% to 95%. CdtB appears to be the most conserved, with 45% sequence identity, even between the least-related CDTs (55). As the active component, CdtB has been demonstrated to share striking similarity with the DNase I protein family (56). At the sequence level, CdtB possesses the essential residues responsible for DNase I enzymatic activities, including residues important in active site and Mg^2+^-binding site (40). At the 3D structure level, CdtB exhibits the canonical characteristics of DNase-like protein: stranded β-sandwich flanked with α-helix and loops (57). Thus, the final destination for CdtB is the cell nucleus, where it can induce DSBs and immediately trigger DNA damage-dependent checkpoint activation (58, 59). Subsequently, stalling of cell cycle progression occurs at G1/S or G2/M transition to block cell division and allow for DNA repair (60).

**Figure 1:**
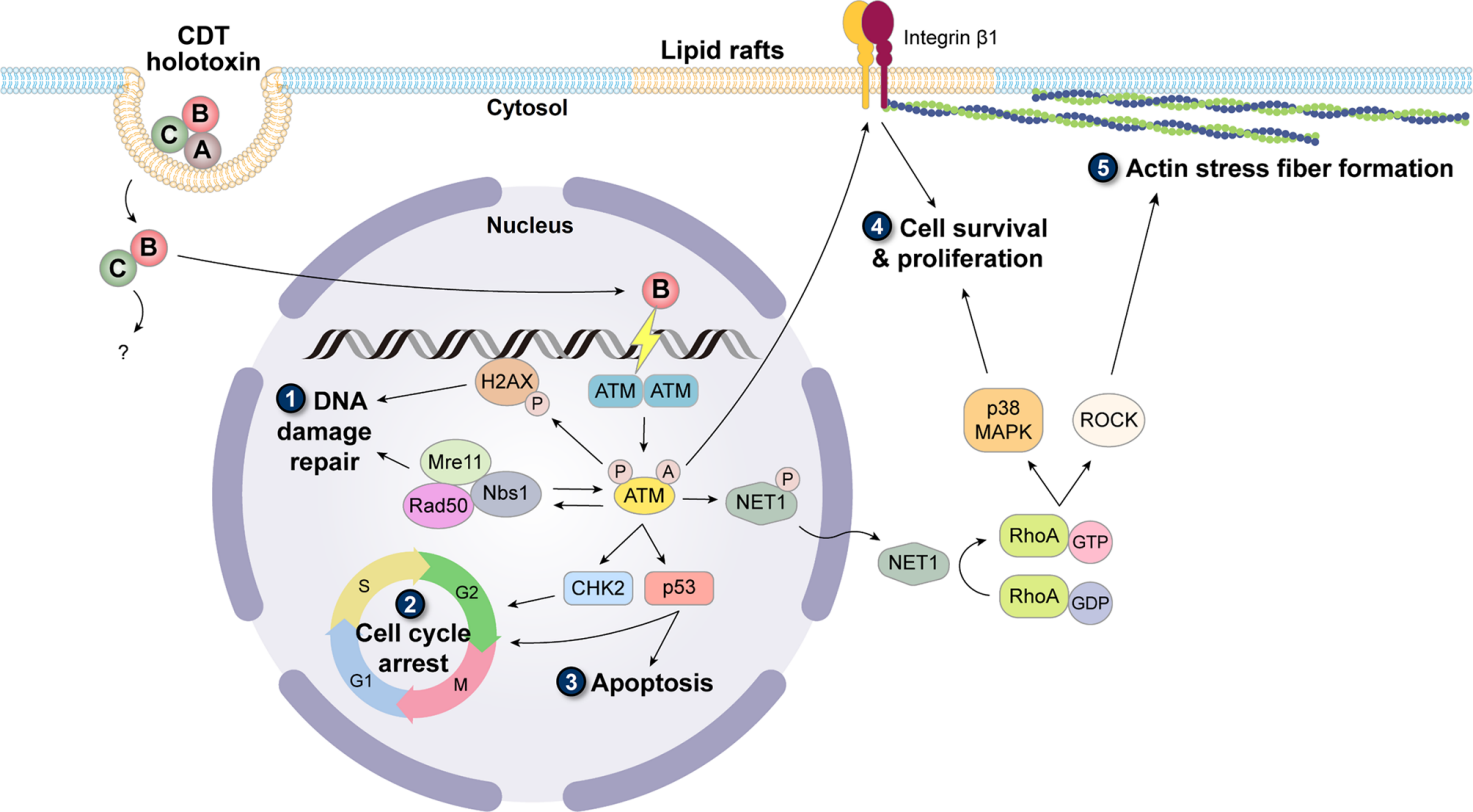
Carcinogenic induction caused by CDT. Binding of CdtA and CdtC to lipid rafts facilitates the entry of CdtB and CdtC. In the cytoplasm, CdtB dissociates with CdtC and translocates into the nucleus alone. As a DNase, CdtB damages the host DNA and immediately triggers the activation of ATM, which is involved in the formation of monomer and biochemical modifications including phosphorylation and acetylation. (1) Activated ATM targets H2AX, phosphorylated on Ser139 (γH2AX), and initiates the cascade of DDR signaling pathway. (2) To allow for DNA repair, with the co-signaling of MRN complex, ATM also activates CHK2 and p53 to stall cell cycle progression. (3) When the DNA damage is too devastating, the cells are prone to undergo apoptosis. However, the cells with misrepaired DNA can continue cycling, thereby accumulating mutations to cause genomic instability. Activated ATM also causes dephosphorylation of Net1, which is activated and translocated to the cytoplasm. Net1 switches the inactive GDP-bound form to the active GTP-bound form of RhoA. The downstream region of RhoA mainly diverges into two pathways: (4) one activates p38 MAPK and further promotes cell survival and proliferation, and (5) the other activates ROCK and induces the formation of actin stress fibers. In addition, stress fibers are often anchored on the focal adhesion complex constituting integrin, of which the inside-out activation signal can be transduced by ATM. Together, these cellular responses triggered by CDT are related to the acquisition of cancer hallmarks.

Binding of CDT holotoxin to the cell membrane primarily depends on CdtA and CdtC. These two subunits adopt a ricin-like lectin structure, forming an aromatic patch and a deep groove on the protein surface, which play key roles in cell surface recognition and association with specific membrane components (61, 62). Despite the specific receptor unidentified, several studies have highlighted the requirement of lipid rafts (sphingolipid- and cholesterol-rich regions on the membrane) for CdtA and CdtC binding to the cell membrane (63–65). Combined with the fact that the deep groove in the holotoxin structure is rather hydrophobic, it is implied that the binding subunits may contain a cholesterol recognition amino acid consensus sequence (CRAC)-like region. Indeed, in further motif analysis, the CRAC site has been identified in CdtC (64, 66). Many molecular mechanisms remain unclear for the subsequent internalization and intracellular transport processes. In addition, it is suggested that these pathways may vary among bacterial species and are also influenced by a broad range of cell types being intoxicated by CDT (67). Nevertheless, a general concept is that after the binding of CDT holotoxin, CdtA remains on the membrane, while CdtB and CdtC are internalized into the cytosol. Only CdtB is delivered to other subcellular compartments and ultimately to the nucleus (56, 68). Upon reaching the nucleus, CdtB exerts its DNase activity to cause DNA damage, which is possibly SSBs with low-dose treatment, and DSBs with high-dose treatment (69). The triggered DDR is dominantly orchestrated by phosphatidylinositol 3-kinase (PI3K)-like protein kinase ataxia telangiectasia mutated (ATM) (70, 71). Activation of ATM simultaneously causes the phosphorylation of histone H2AX (γH2AX) as well as the recruitment of Mre11-Rad50-Nbs1 (MRN) complex, which provides a platform for DNA repair, and sets off checkpoint responses *via* the phosphorylation of CHK2 and p53, resulting in cell cycle arrest thus inhibiting cell proliferation (**Figure 1**) (72). In most cases, DNA damage becomes way too devastating, and the repair system fails to rescue the situation, which consequently leads to cell death (73, 74) or senescence (75–77). However, in a few cases the intoxicated cells bypass death; these cells escape the built-in carcinogenesis barrier of cell death/senescence and are likely to develop a tendency for cancer formation, including genomic instability, heightened mutation frequency, and anchorage-independent cell growth (13, 78, 79).

## Genotoxicity and Cancer Development

Since the discovery of genotoxin, its DNA-damaging activity has long been considered a powerful cell-killing strategy. However, in recent years, its role in pathogenesis has started to appear in a completely different perspective. The connection between genotoxins and cancer has been assessed through both gut microbiota analysis and epidemiology profiling. The research revealed a higher prevalence of *cdt* and *pks* (gene encoding colibactin)-positive *E. coli* in the gut microbiome of patients with inflammatory bowel disease and CRC than in the non-cancer group (26, 80). Moreover, a mutational pattern characteristic of colibactin exposure was found to be enriched in the sequencing data of two independent cohorts of primary CRC tumors and CRC metastases (81). Several *in vivo* studies also validated the potential of genotoxin to increase the risk of malignancy. A/JCr mice developed hepatic dysplastic nodules after chronic infection with *Helicobacter hepaticus* (34). *H. hepaticus* infection causes chronic hepatitis; however, the progression of inflammation toward dysplasia was found to be associated with the presence of CDT, which upregulates a subset of proinflammatory mediators, and increases hepatocyte proliferation as well as mRNA expression of anti-apoptotic proteins (34). Moreover, invasive carcinoma can be detected in susceptible mice exposed to *H. hepaticus* but not in those exposed to the isogenic *cdtB* mutant (35). The study partly explained that CDT affects Stat-3 signaling, thereby promoting oncogenic processes. Similarly, persistent infection with CDT-harboring *C. jejuni* resulted in tumor formation in *Apc^Min/+^* mice fed with 1% dextran sulfate sodium (DSS) (28). The developed tumor number and tumor size were significantly reduced when the infecting bacteria possessed mutated *cdtB*. Additionally, human colonic epithelial cells with defective genes commonly observed in CRC models are prone to micronucleus formation and anchorage-independent cell growth after CDT treatment (79). Collectively, these findings indicate that the cell response to genotoxin intoxication appears to be detrimental, but not necessarily destructive.

To elucidate the detailed mechanisms behind this phenomenon, numerous studies have been conducted in recent years. Generally, CDT-intoxicated cells tend to enter cell cycle arrest as soon as the DNA damage takes place; however, a proportion of cells that manage to tolerate DNA damage induced by CDT and persist cycling have been identified, and further analysis indicated that these cells showed signatures of malignant transformation (13). As a consequence of dampened DDR and the slowing of replication fork velocity, the genetic stability and integrity are disrupted, which was observed through elevated fragile sites expression and chromosome aberrations (13, 82). As DNA lesions continue to accumulate, they increase the risk of mutation occurrence and are more likely to lead the cells down the path of pro-cancerous progression. Additionally, unrepaired DNA lesions can lead to micronuclei formation after cell division, which causes a proinflammatory response once micronuclei are sensed as cytosolic DNA and triggers the cGAS-STING pathway (83).

On the other hand, the master regulator of CDT-triggered DDR, ATM, transduces not only the DNA repair signal, but also activates a survival pathway involving p38 mitogen-activated protein kinase (MAPK) and integrin β1 (13, 78, 84). As its name indicates, CDT causes the distension morphology of the intoxicated cells, which has been examined to be associated with the formation of actin stress fiber (85). This phenomenon raised the possibility that there might be an intriguing crosstalk between DNA damage and cytoskeleton arrangement. Later studies identified a small GTPase (RhoA) as a crucial molecule in this potential signaling pathway (59). RhoA mainly participates in the coordination of actin cytoskeleton reorganization and focal adhesion, which may contribute to tumor invasion and metastasis (86). It can be activated by a nuclear-localized guanine nucleotide exchange factor (GEF) Net1 (87), the activation of which requires dephosphorylation at the inhibitory site Ser152 (88). The detailed molecular mechanism of how the Net1/RhoA response is triggered remains obscure; however, the participation of ATM and flap structure-specific endonuclease 1 (FEN1) has been implied (59, 89). The downstream pathway of RhoA diverges into p38 MAPK and Rho-associated kinase (ROCK) signaling. Aside from a plethora of reports concerning the proinflammatory effect of p38 MAPK (90–92), it has also been reported that sustained p38 MAPK is vital for cell survival under genotoxic stress (13, 93, 94). In parallel, ROCK signaling manipulates the stress fiber formation and cellular contractility (95). Moreover, ATM signaling can act as an inside-out activation signal for integrin β1, a membrane-bound receptor that transduces the signal favoring cell survival and proliferation. Accordingly, abolishing this signaling pathway compromises the ability of the intoxicated cells to avoid anchorage-independent cell death (78).

Furthermore, a recent study showed that disruption of the intestinal structural barrier facilitates dissemination of the gut bacteria, which can be delivered to the liver through intestinal capillaries and the portal vein. Bacteria in the liver recruit immune cells and promote the formation of an inflammatory environment, likely establishing a premetastatic niche (96). This phenomenon suggests a role for CDT in the process of metastasis. Theoretically, upon infection with CDT-harboring bacteria and the secretion of CDT, a group of cells become intoxicated by CDT. Most of these cells die, which damages the integrity of the intestinal barrier, whereas a small proportion survives; these cells become tumor cells and travel with the bacteria to the liver. In the inflammatory environment promoted by the bacteria, tumor cells settle in the premetastatic niche and, thus, favor distant metastasis formation.

## Conclusions and Future Perspectives

Extensive studies have explored how CDT has been linked to a variety of diseases, and most of them reported the proinflammatory nature of this special bacterial genotoxin, which potentiates the carcinogenic property of CDT. CDT-induced genotoxic stress not only fuels the inflammatory response but also disrupts the structural barrier by inducing epithelial cell death. However, a small portion of the intoxicated cells outrun cell cycle arrest and continue to proliferate with incorrectly repaired or unrepaired DNA lesions. As more DNA lesions accumulate, it enhances the mutation frequency, interferes with genomic stability, and develops tumor initiation. The connection between the infection of CDT-harboring bacteria and cancer development has been reported in several animal studies, which demonstrated that with the help of CDT, bacteria-induced inflammatory response can be further depraved to malignancy formation (summarized in **Figure 1**).

This review emphasized the importance of recent findings regarding the genotoxicity of CDT associated with cancer formation. However, the direct link between toxin action and intracellular delivery and its clinical relevance remains largely unclear. Various crucial issues must be addressed: (i) Whether long-term persistent infection of CDT-producing bacteria is related to an increased risk of cancer progression in the host should be evaluated in clinical cases. (ii) The concentration of genotoxin produced by bacteria that can naturally cause oncogenesis *in vivo* is unclear. (iii) Although delivery of CdtB into the nucleus and triggering of DNA damage have been demonstrated, the molecular mechanism and intracellular trafficking pathways of the various CDTs produced by different bacterial species remain to be clarified. It is crucial to explore the detailed mechanism of CDT function using *in vivo* models or in clinical studies. Further investigations are required to provide pivotal insights into the mechanisms underlying the interplay between genotoxicity and cancer development. This may aid in developing novel strategies to combat diseases caused by pathogens along with their virulence factors.

## Author Contributions

Conception or design of this work: C-HC, M-LK, and C-HL. Writing the manuscript: Y-RL, Y-FC, and JM. Y-RL and JM were equally contributed to this work. All authors contributed to the article and approved the submitted version.

## Funding

This research was supported by Taiwan Ministry of Science and Technology (108-2911-I-005-509, 109-2911-I-005-503, 109-2320-B-182-025-MY3, and 109-2320-B-182-029-MY3), Chang Gung Memorial Hospital (CMRPD1I0061-3, CMRPD1J0021-3, CMRPD1K0361, and BMRPE90), and Tomorrow Medical Foundation.

## Conflict of Interest

The authors declare that the research was conducted in the absence of any commercial or financial relationships that could be construed as a potential conflict of interest.

## Publisher’s Note

All claims expressed in this article are solely those of the authors and do not necessarily represent those of their affiliated organizations, or those of the publisher, the editors and the reviewers. Any product that may be evaluated in this article, or claim that may be made by its manufacturer, is not guaranteed or endorsed by the publisher.
